# Enhancing Agricultural Sustainability by Improving the Efficiency of Lignocellulosic Biomass Utilization in the Ruminant Diet via Solid-State Fermentation with White-Rot Fungi: A Review

**DOI:** 10.3390/microorganisms13071708

**Published:** 2025-07-21

**Authors:** Qi Yan, Osmond Datsomor, Wenhao Zhao, Wenjie Chen, Caixiang Wei, Deshuang Wei, Xin Gao, Chenghuan Qin, Qichao Gu, Caixia Zou, Bo Lin

**Affiliations:** 1 College of Animal Science and Technology, Guangxi University, Nanning 530004, China; 2318401013@st.gxu.edu.cn (Q.Y.); 2418301049@st.gxu.edu.cn (W.Z.); 2418391007@st.gxu.edu.cn (W.C.); 2218301034@st.gxu.edu.cn (C.W.); 2218301033@st.gxu.edu.cn (D.W.); 2118391005@st.gxu.edu.cn (X.G.); 2318391044@st.gxu.edu.cn (C.Q.); guqichao@st.gxu.edu.cn (Q.G.); 2 Guangxi Key Laboratory of Animal Breeding, Disease Control and Prevention, Nanning 530004, China; 3 Science and Technology Backyard of Guangxi Fusui Dairy Industry, Chongzuo 532100, China; 4 College of Animal Science and Technology, Jilin Agricultural University, Changchun 130117, China; datsomorosmond@gmail.com

**Keywords:** agricultural by-products, preprocessing, roughage ingredients, ruminant ration, livestock production

## Abstract

Against the backdrop of the green circular economy, the exploration of reliable and sustainable applications of lignocellulosic biomass (LCBM) has emerged as a critical research frontier. The utilization of LCBM as a ruminant roughage source offers a promising strategy to address two pressing issues: the “human-animal competition for food” dilemma and the environmental degradation resulting from improper LCBM disposal. However, the high degree of lignification in LCBM significantly restricts its utilization efficiency in ruminant diets. In recent years, microbial pretreatment has gained considerable attention as a viable approach to reduce lignification prior to LCBM application as ruminant feed. White-rot fungi (WRF) have emerged as particularly noteworthy among various microbial agents due to their environmentally benign characteristics and unique lignin degradation selectivity. WRF demonstrates remarkable efficacy in enzymatically breaking down the rigid lignocellulosic matrix (comprising lignin, cellulose, and hemicellulose) within LCBM cell walls, thereby reducing lignin content—a largely indigestible component for ruminants—while simultaneously enhancing the nutritional profile through increased protein availability and improved digestibility. Solid-state fermentation mediated by WRF enhances LCBM utilization rates and optimizes its nutritional value for ruminant consumption, thereby contributing to the advancement of sustainable livestock production, agroforestry systems, and global environmental conservation efforts. This review systematically examines recent technological advancements in WRF-mediated solid-state fermentation of LCBM, evaluates its outcomes of nutritional enhancement and animal utilization efficiency, and critically assesses current limitations and future prospects of this innovative approach within the framework of circular bioeconomy principles.

## 1. Introduction

A complex biopolymer known as lignocellulose comprises interlinked cellulose, hemicelluloses, and lignin compounds. Lignocellulosic biomass (LCBM), a term for lignocellulose in plant dry matter, is composed of substances with unique physical and chemical properties. The production of LCBM is enormous, especially from agricultural production, and so vast that it is difficult to measure accurately. Between 2000 and 2022, global production of major crops (cereals, sugar crops, vegetables, oilseeds, fruits, roots and tubers, and others) was 9.6 billion tonnes, corresponding to a 56 percent increase. Of these, sugarcane (20% of the total, 1.9 billion tonnes), maize (12%, 1.2 billion tonnes), and wheat and rice (8%, 0.8 billion tonnes each) were prominent [1]. It is forecasted that with increasing agricultural production, the production of LCBM, such as crop residues, would reach about 200 billion tonnes [2]. In the context of sustainable development, it has become particularly urgent to solve the problems of environmental damage and resource waste caused by lignocellulosic biomass, as the current mode of global economic growth has transformed from rapid development to long-term sustainable development [3]. Concurrently, global concern for sustainable energy and environmental protection has increased rapidly. Practically, if the large amount of LCBM produced through agricultural production is effectively utilized, it can alleviate the pressures associated with energy depletion and environmental protection issues [4].

Ruminants are naturally the best utilizers of LCBM, digesting and utilizing it through the action of microbiota inhabiting the rumen, which is one of the most potent plant fiber-digesting organs known [5]. Successful cases of ruminants utilizing LCBM as a source of dietary roughage have been widely reported [6]. Including LCBM as part of roughage and utilization by ruminants offers a lot of advantages, such as lowering farming costs, reducing the area of arable land under pasture cultivation, and solving environmental problems caused by LCBM. However, LCBM may reduce ruminants’ growth, efficiency, and productivity. Firstly, the tight mesh structure of cellulose, hemicellulose, and lignin in the cell wall of LCBM makes it difficult for rumen microorganisms to attach effectively, enzymatically hydrolyze/digest, and utilize nutrients in native (untreated) LCBM. Secondly, ruminants cannot utilize the large amount of lignin in LCBM; even if they could, lignin is a non-nutritive, inert substance, and as such, only a small amount of LCBM is utilized in ruminant diets [7].

The use of different technical means to improve the efficiency of LCBM utilization in ruminants has been widely reported in recent years. Numerous excellent papers have also reviewed these technical tools [8,9]. Among them, white-rot fungi (WRF) have received the most attention due to being environmentally friendly (non-polluting) and lignin degradation selectivity. Many researchers have carried out studies to improve the utilization efficiency of LCBM (involving rice straw, tea by-products, etc.) using WRF solid-state fermentation. WRF is effective in enzymatically disassembling the tight lignin, cellulose, and hemicellulose junctions within the cell walls of LCBM, reducing lignin content (largely indigestible by ruminants) and increasing the quality of other nutrients (e.g., proteins and easily digestible carbohydrates). Van Kuijk et al. [7] and Nazri Nayan et al. [10] previously provided excellent overviews of their teams’ work, as well as helping to advance the field of solid-state fermentation using various LCBM and WRF. However, to the best of our knowledge, there is a lack of a review that systematically summarizes the organization of research on solid-state fermentation of LCBM by WRF, key parameters to successful fermentation (strains, substrates, and environmental factors), and animal experiments (in vivo, in vitro, and in situ) in ruminants. Furthermore, there have been some technological advances in the field in recent years, and the current literature needs to be updated.

To begin with, this review will focus on analyzing the characteristics of the use of LCBM in ruminant farming (benefits to the circular economy) to illustrate the necessity of employing WRF in the solid-state fermentation of LCBM. Secondly, the latest technological progress of WRF solid-state fermentation of LCBM will be described and analyzed, particularly in terms of non-sterilization of substrates and enhancement of WRF solid-state fermentation efficiency. Subsequently, the effects of WRF solid-state fermentation on LCBM are described and analyzed (examined through chemical components, animal experiments, etc.). Finally, the contribution of using WRF solid-state fermentation of LCBM to the sustainable development of agriculture is initially described and analyzed (mainly WRF solid-state fermentation products have the potential to reduce methane production in ruminants, and WRF solid-state fermentation improves the efficiency of LCBM utilization). This review will provide a reference for the future focus of WRF solid-state fermentation of LCBM, thus contributing to the improvement of the utilization efficiency of LCBM in ruminants and the sustainability of agricultural production.

## 2. Global Research Trends

Analyzing the keywords of studies related to LCBM solid-state fermentation utilizing WRF helps in understanding the focus of the research and the latest trends [11,12]. This review collected studies associated with the application of WRF solid-state fermentation technology for the pretreatment of LCBM in ruminant diets from 1 January 2000 to 2025 using the Web of Science and NCBI Pubmed databases. An in-depth search of the published scientific literature was conducted using the following search terms. Search terms included the following: TS = ((“White rot fungi” or “White rot fungal” or “White rot basidiomycete” or “White rot fungus”) and (“degrade” or “treatment” or “solid-state fermentation” or “non-aqueous fermentation” and (“ruminant” or “cow” or “cattle” or “bovine” or “sheep” or “ovine” or “goat” or “caprine” or “mare”)). In total, 90 original research articles and 6 review articles were collected for subsequent analysis.

**Figure 1:**
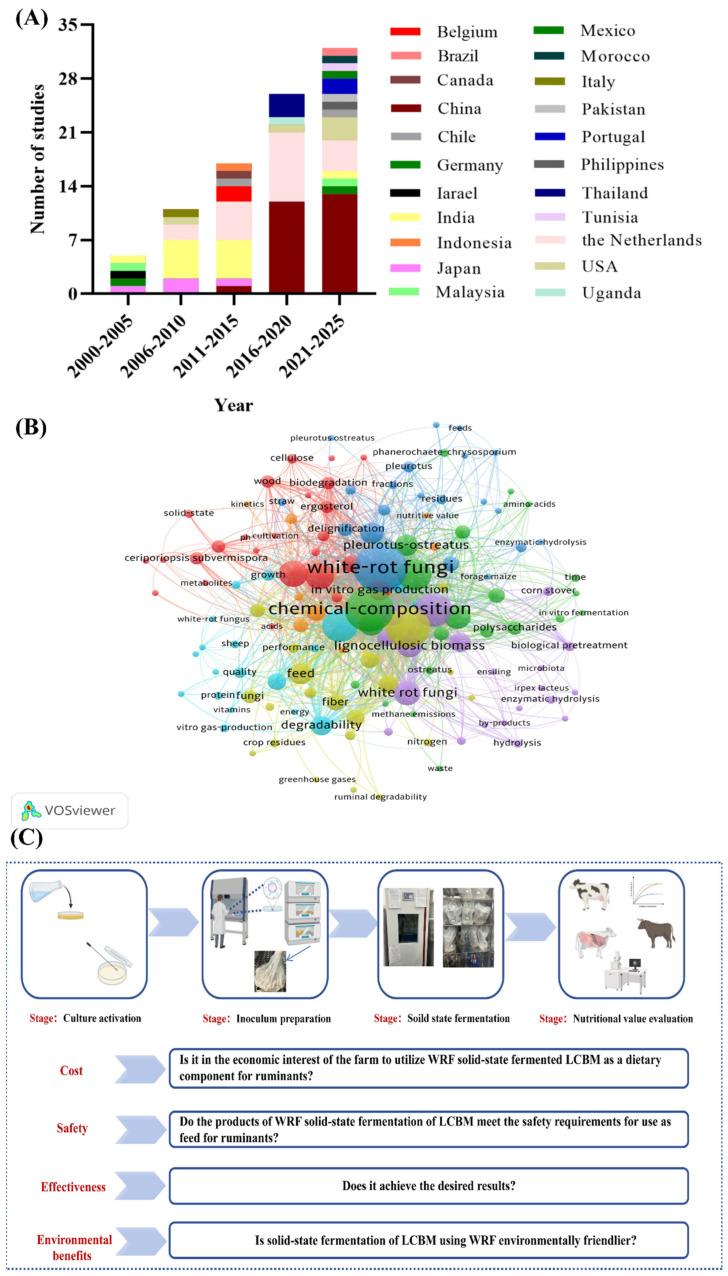
Basic guidelines and approximate flow of LCBM for solid-state fermentation using WRF. (**A**) Distribution of countries studied on solid-state fermentation of LCBM using white-rot fungi, 2000–2025; (**B**) Keyword co-occurrence map of studies on solid-state fermentation of LCBM using white-rot fungi, 2000–2025; (**C**) Basic principles of solid-state fermentation of LCBM using white-rot fungi.

Because of the decreasing global per capita arable land area, environmental damage is becoming more and more prominent, which has induced a strong interest in recyclable economy and recyclable agricultural production. From 2000 to 2025, the number of research departments utilizing WRF solid-state fermentation LCBM is increasing (Figure 1A). Of these, the Netherlands is the country that has consistently provided a large amount of research for the WRF solid-state fermentation LCBM. Remarkably, China is the country that has seen a surge in the number of studies in recent years. The increase in the number of studies in China can be attributed to the increasing scale of ruminant farming due to the amount of meat and dairy products consumed by consumers, while the cost of feed remains high [13,14,15,16,17,18]. Keyword analysis is able to analyze the focused concerns in the field. It is easy to see from (Figure 1B) that chemical composition and white rot fungi are currently the biggest concern (according to the bubbles and font size shown in the figure). This is because it is relatively easy and affordable to analyze the feeding value of reactive fermentation products using laboratory chemistry. The high frequency of studies using in vitro rumen gas production can also be attributed to the same reason as laboratory chemical analysis. Laboratory chemical analysis and in vitro rumen gas production are more affordable and less labor-intensive for animal feeding experiments. To carry out research in a particular field, it is important to clarify the research purpose of the field. Solid-state fermentation of LCBM using WRF is the primary research objective. To improve the efficiency of utilization of LCBM as a component of ruminant diets. The utilization of WRF solid-state fermentation LCBM can be briefly divided into four steps (Figure 1C). The four main principles to be considered are the following: low cost, safety in compliance with standards, the effectiveness or feeding value of the solid-state fermentation, and whether or not it reduces the pressure of environmental pollution.

## 3. Pros and Cons of Utilizing LCBM in Ruminants

**Figure 2:**
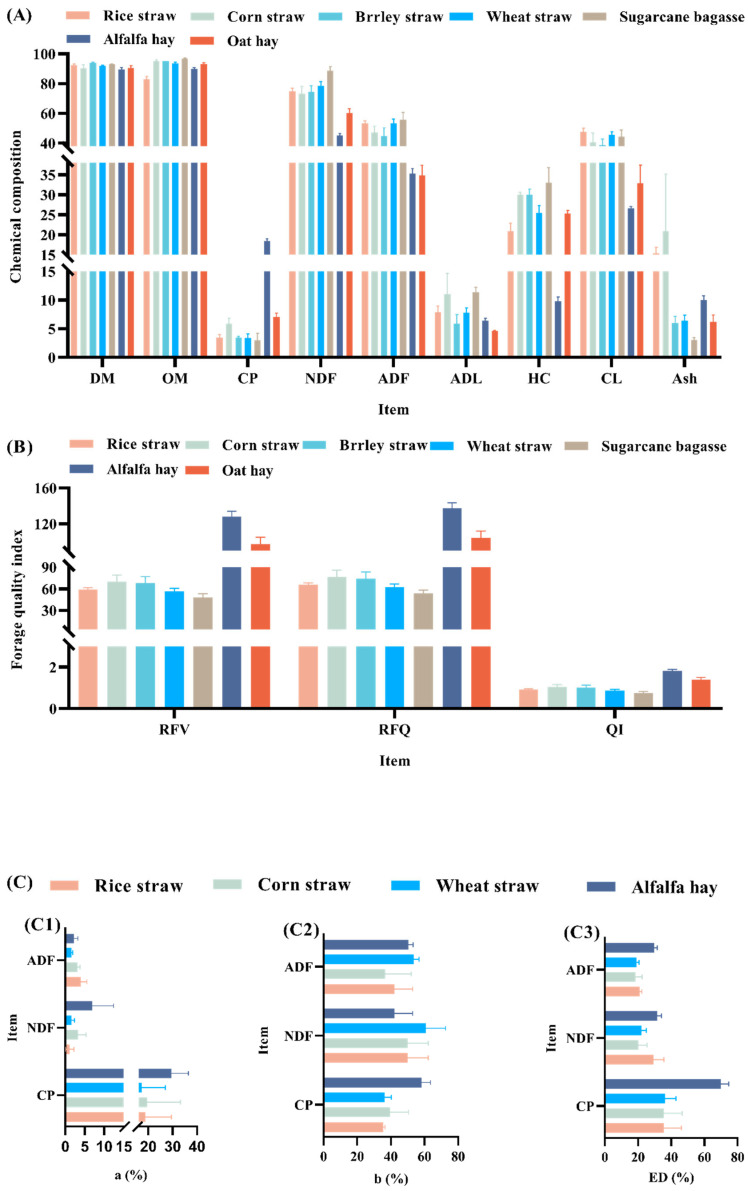
Comparison of nutritive value, nutrient index, and rumen degradation parameters of several LCBM with alfalfa and oat hay [25,26,27,28,29,30]. (**A**) Reported chemical composition of LCBM, alfalfa hay, and oat hay (calculations based on research data); (**B**) Forage quality indices of LCBM, alfalfa hay, and oat hay (calculations based on research data; calculation formula reference [31,32]); (**C**) In situ rumen degradation parameters for LCBM and nutrients in alfalfa hay (%); a, immediately degradation at time (%); b, potentially degradable fraction (%); ED, effective degradability (%); DM, dry matter; OM, organic matter; HC, hemicellulose; CL, cellulose.

There are two reasons for ruminants to be connoisseurs of utilizing LCBM: special rumination/regurgitation mechanisms and the development of the rumen, a key digestive organ. The first is due to the process of rumination, which is simply the separation of large particles from small ones via density screening in the foregut, followed by the return of large particles to the mouth for further chewing and re-entry into the intestine [19]. This process increases the surface area of the chyme through repeated chewing and promotes its degradation in the rumen. Another vital reason is possessing a unique organ, the rumen, which has evolved to adapt to the survival environment. The rumen is inhabited by microbiota such as bacteria, fungi, and protozoa, which help ruminants degrade LCBM via degradation mechanisms such as enzymolysis (primarily involving cellulases and xylanases) [20,21]. Overall, ruminant rumination behavior and the function of the rumen in processing LCBM constitute a complex process that will not be discussed here. Figure 2A summarizes some research information on the nutritional value of LCBM versus alfalfa hay and oat hay (high-quality forage for ruminants). It is easy to see that the crude protein (CP) content of alfalfa hay (18.46%/DM) was much higher than that of LCBM, and the acid detergent lignin (ADL) content (6.44%/DM) was lower than that of LCBM. This indicates that the available nutritive value of alfalfa hay is higher than that of LCBM. Similarly, oat hay demonstrated higher available nutrient values than LCBM. Cell wall components, namely neutral detergent fiber (NDF), acid detergent fiber (ADF), and ADL, were much higher in LCBM than in alfalfa hay versus oat hay. This affected the availability and degradability of LCBM to microorganisms in rumen [22]. Lignin, a cell wall component, cannot be degraded under anaerobic conditions in the rumen and is an unavailable non-nutrient for ruminants. Secondly, lignin forms a tightly linked structure encapsulating cellulose and hemicellulose, hindering the accessibility of hydrolytic enzymes secreted by rumen microorganisms [7].

Figure 2B shows that the relative feed value (RFV) of LCBM was in the range of 48.14–69.93, whereas the RFV of alfalfa hay of 128.16 and oat hay of 97.01 were much higher than that of LCBM. Similarly, the results of relative feeding quality (RFQ) and quality index (QI) were similar to those of RFV, which were much higher for alfalfa hay and oat hay than for LCBM. The above shows that ruminants can utilize LCBM as a part of the feed component. However, the nutritional availability of LCBM for ruminants is much lower than that of conventional forages (e.g., alfalfa hay and oat hay) [23]. The in situ rumen technique is one of the experimental tools used to visualize and economically respond to the dynamics and final outcome of the rumen fiber degradation [24]. Figure 2C shows the results of studies related to in situ rumen degradation between LCBM and high-quality forages. It is easy to see that LCBM has a lower degradation rate than alfalfa hay in the nutrient a-part (fast degradation part in the rumen) and ED (effective degradability fractions of CP, NDF, and ADF). The need to utilize white-rot fungi to improve the nutritional value of LCBM is further highlighted.

## 4. Key Technical Factors for LCBM in Solid-State Fermentation at WRF

Currently, research on the solid-state fermentation of LCBM utilizing white-rot fungi is mainly focused on laboratory scale, and there are few reports on large-scale processing. Parameter variables affecting the solid-state fermentation of LCBM by white-rot fungi can be divided into substrate, fungal, and environmental parameters. According to previous reports, changing the parameter variables in solid-state fermentation will significantly affect the growth and metabolism of fungi, thereby affecting the quality of the final fermented LCBM.

### 4.1. Fungal

Screening for the optimal WRF for different species of LCBM is essential. Numerous studies have confirmed the selective adaptation between WRF and LCBM, with differences in the effectiveness of the same fungus on the solid-state fermentation of different LCBM. Zuo et al. (2019) selected seven different WRFs for the solid-state fermentation of two varieties of corn stover, among which *Irpex lacteus* was the best fermenting WRF, which reduced the ADL content of the two varieties by 33.0% and 40.0%, respectively [33]. Secondly, Niu et al. (2020) selected eight varieties of wheat straw for solid-state fermentation using *I. lacteus*, and the degradation of ADL ranged from 517.7 g/kg DM to 814.3 g/kg [34]. The results of the above studies showed that the solid-state fermentation of different varieties of the same species of LCBM by the same strain of WRF yields different outcomes. For the adaptation of fungi to LCBM, further screening for efficient strains is needed.

### 4.2. Substrate

The choice of substrate can be associated with the natural growth environment of WRF. The starting point of using WRF in the solid-state fermentation of LCBM is to simulate the natural growth environment of WRF [35]. WRF are widely distributed in nature and mainly occurs in softwood and hardwood rot in forests. At present, LCBM reported in this field has a wide range, but primarily focuses on bulk food crop straws such as rice straw, wheat straw, and corn straw. The high yield is the most important reason for choosing bulk grain crops for solid-state fermentation. Secondly, bulk food crop residues are easier to collect in the field. In addition, WRF can utilize these crop residues for growth and development. The above crop residues are the most common roughage used in ruminant production. However, it does not mean that other LCBMs are not available and interesting to study; it just highlights that these crop straws are more exemplary.

Solid-state fermentation is fermentation in the absence of free water and with very low levels of free water. Solid-state fermentation usually has a moisture content between 30 and 85%; the right moisture content is essential for mundane fermentations. Lower moisture levels reduce the flow of nutrients and metabolites to the microorganisms, affecting fungal growth. Higher moisture levels reduce the porosity of the substrate, limiting the flow of oxygen and heat, affecting microbial growth, and increasing the risk of contamination [36]. Table 1 shows that the moisture content in the reports was controlled and ranged between 70 and 85%, which is in line with the appropriate moisture interval for solid-state fermentation. Some studies have taken an approach of directly submerging all the LCBM in water, which is ineffective in controlling moisture content and may cause uncontrollable predicaments in subsequent fermentation.

Pretreatment of LCBM prior to selecting WRF for solid-state fermentation of LCBM can improve the efficiency and stability of the fermentation. Autoclaving is routinely practiced for substrate pretreatment, and autoclaving has become an almost mandatory processing step for studying LCBM in solid-state fermentation utilizing WRF. However, the energy consumption required for autoclaving is not in line with the central theme of the green circular economy. In addition, the complex operation of autoclaving is not practical for large-scale and small-scale farmers, and a new means to replace autoclaving is urgently needed [7]. The search for alternatives to autoclaving step has become a research priority for scholars in this field. Modern alternative research ideas mainly focus on chemical methods like acid and alkaline treatments. Niu et al. reported the effect of silage as an alternative to autoclave pretreatment of wheat straw on the efficacy of solid-state fermentation of wheat straw with WRF. The results showed that *Irpex lacteus* was able to dominate the ecological niche after silage, selectively degrading lignin by 28% and increasing rumen dry matter digestibility (IVDMD) by 18% [37]. Zheng et al.’s study followed the same idea as Niu et al.’s two-stage fermentation (silage of crop straw followed by inoculation of WRF for solid-state fermentation). The difference was that Zheng et al. added different percentages of rumen liquid during the silage stage to better acidify the naked oat straw, and the results showed that using 3% rumen liquid for silage first, followed by inoculation with *I. lacteus* for solid-state fermentation, reduced the lignin of naked oat straw by 44% [38]. Abubakar Sufyan et al. used a large batch (8400 kg) of wheat straw soaked in a lime solution for 12 h and then inoculated with *Pleurotus ostreatus* for solid-state fermentation [39]. After 30 d, the lignin content of wheat straw was reduced by 30%, in vitro dry matter digestibility (IVDMD) increased by 27%, and mycotoxins were below 5 μg/kg. The above method is operable for pastures and farmers and is an effective alternative to autoclaving.

### 4.3. Environmental Parameters

Temperature is a key factor affecting the efficiency of solid-state fermentation (SSF), as microbial growth and development are often temperature-sensitive, affecting the production of enzymes or metabolites [40]. Table 1 shows that 24–30 °C was the common incubation temperature in this study, and 24–28 °C was the most common incubation and fermentation temperature. However, a single study of external environmental incubation temperatures may not be able to fully characterize the response of WRF to temperature during solid-state fermentation. Aerobic fermentation is a process that generates heat, and in another type of fermentation (silage), a great deal of research has been carried out on the cumulative temperature of fermentation. Studies have shown that the higher the temperature during fermentation, the higher the dry matter loss in the fermented material [41]. The influence of the cumulative temperature of fermentation on the WRF solid-state fermentation process is still unexplored or inadequate, as only a few/limited studies have been carried out in this area. In addition, there still exists a knowledge gap as to whether different substrate particle sizes and stacking densities affect the cumulative temperature of fermentation and thus lead to differences in fermentation results.

Regardless of the research and application purposes (treatment of LCBM, treatment of hospital wastewater, treatment of microplastics, etc.), the rationale for selecting WRF is basically using its extracellular enzymes (VP, Lip, Mnp, Lac, etc.). Therefore, research focus has also been on how to regulate enzyme activity through exogenous inducers. Among the most popular inducers are Mn and Cu. These are essential micronutrients for fungal growth, acting as a metal activator of fungal enzymes like oxidases. The effects and mechanisms of Mn and Cu on WRF ligninolytic enzymes are well summarized in the review by Viviana Benavides et al., and we will not dwell on them here [42]. However, suppose the ultimate purpose of adding Mn and Cu to the WRF solid-state fermentation of the LCBM system is to feed ruminants. In that case, it is recommended that they be added in the form of CuSO_4_, MnSO_4_, CuSO_4_, and MnSO_4_ (common forms found in ruminant dietary supplements), and that attention be given to the amount of added (add as little as possible).

Fermentation time is critical to the effectiveness of WRF solid-state fermentation of LCBM, and achieving the desired fermentation effect as early as possible can reduce the loss of easily digestible material from LCBM for ruminants. Table 1 shows that the time of WRF solid-state fermentation of LCBM varies greatly from 7–63 d for experiments conducted by different researchers. The current lack of research on fermentation time in this field is an approximation time, as differences in fermentation effects due to operational differences may exist in batch application situations. The difference in the effect of fermentation may lead to the meaninglessness of precise fermentation time. In other words, approximate time ranges, rather than precise times, are currently the most needed regarding fermentation times in application situations. Within the approximate timeframe, the operator can sample the fermentation to effectively assess the effect of the fermentation and determine whether or not to terminate the fermentation. In addition, the method of terminating the fermentation and the stability after opening the bag needs to be studied.

**Table 1:** Fermentation parameters of WRF solid state fermentation LCBM.

Strain/Straw	Size (cm)	Temperature (°C)	Humidity (%RH)	Strain Additions	Time (day)	Moisture (%)	Sterilization Conditions	Ref.
Wheat straw								
*P. ostreatus*	5–10	25	78	4%	21	infuse	100 °C (1 h)	[43]
*P. ostreatus* and *T. versicolor*	1.5–2	30	-	0.5%	30	30%	121 °C (15 min)	[44]
*C. subvermispora* and *L. edodes*	0.5	24	-	10% (Barley grains)	39 and 52	40%	121 °C (1 h)	[45]
*P. chrysosporium*, *P. ostreatus* and *I. lacteus*	0.1	28	-	3 agar	7, 14, 21, and 49	70%	121 °C (20 min)	[46]
*P. ostreatus*	2.5	24	75–85	3%	0, 10, 20, and 30	70%	Lime, steam, and formaldehyde sterilization	[47]
Rice straw								
*P. ostreatus*	2–3	25	75–80	3% (millet grain)	30	50%	121 °C (25 min)	[48]
*P. chrysosporium* and *P. ostreatus*	2–3	25	75–80	5% (millet grain)	30	infuse	121 °C (45 min)	[49]
*P. ostreatus*	2–3	22	80–90	3–5%	35	infuse	Alternative silage sterilization	[50]
*C. subvermispora*, *L. edodes*, *P. eryngii* and *P. ostreatus*	2–5	24	75	6 g	21 and 42	infuse	121 °C (1 h)	[51]
*C.subvermispora, L. edodes* and *P. eryngii*	3–5	24, 30, 35, and 40	-	5%	28, 42, 49, and 56	75%	121 °C (1 h)	[52]
Corn straw								
*P. ostreatus*	2	24	70	1%	21, 28, and 35	-	121 °C (1 h)	[53]
*L. edodes* and *P. eryngii*	3	24	70	2.5%	21, 42, and 63	-	121 °C (1 h)	[54]
*P. ostreatus*, *L. edodes*, *H. erinaceus*, *P. eryngii*, and *F. filiformis*	2–3	24	75	8%, 10%	14, 21, 28, 35, and	-	121 °C (2 h)	[55]
*P. diamor*, *P. eryngii*, *P. sajor-caju* and *P. citrinopileatus*	2–3	25	70–80	10%	21	-	121 °C (1 h)	[56]
*C. subvermispora*, *L. edodes*, *P. eryngii*, and *P. ostreatus*	2–5	24	75	6 g	21 and 42	infuse	121 °C (1 h)	[51]
Oher straw								
*P. chrysosporium*, *C. subvermispora*, *L. edode*, and *P. acerina*/Canola straw	1–2	25	-	3%	10, 20, and 30	50%	121 °C (15 min)	[57]
*A. bisporus*, *P. djamor*, *C. indica*, and *P. ostreatus*/Bagasse	2	24	75–85	3%	21 and 56	75%	90 °C (2 h)	[58]
*L. edodes*, *P. eryngii*, and *P. citrinopileatus*/Grage stalk	0.3	28	85	2.5%	28, 35, and 42	infuse	121 °C (30 min)	[59]
*P. citrinopileatus*/Cowpea straw	2	28	75	4%	22	75	121 °C (30 min)	[60]
*P. ostreatus* and *P. chrysosporium*/White tea straw	2–3	25	60	6%	7, 14, 21, and 28	70	121 °C (20 min)	[61]

Note: This table summarizes some of the studies with complete fermentation conditions, including size and length of substrate. *P. ostreatus*, *Pleurotus ostreatus*; *T. versicolor*, *Trametes versicolor*; *C. subvermispora*, *Ceriporiopsis subvermispora*; *L. edodes*, *Lentinula edodes*; *I. lacteus*, *Irpex lacteus*; *P. eryngii*, *Pleurotus eryngii*; *H. erinaceus*, *Hericium erinaceus*; *P. chrysosporium*, *Phanerochaete chrysosporium*; *P. diamor*, *Pleurotus djamor*; *Pleurotus sajor-caju*; *P. citrinopileatus*, *Pleurotus citrinopileatus*; *A. bisporus*, *Agaricus bisporus*.

## 5. Effects of WRF Solid-State Fermentation of LCBM

### 5.1. Chemical Nutrient Composition of LCBM

The chemical nutrient composition of LCBM is significantly altered through WFR solid-state fermentation. As shown in Table 2, the available nutrient values of LCBM, which are usually fermented, were all enhanced. Most studies have shown that the DM, NDF, ADF, ADL, HC, CL, and OM content of LCBM decreased at the end of solid-state fermentation. This is because WRF needs to utilize the nutrients in LCBM for its growth. The key objective of solid-state fermentation of LCBM using WRF is the resolution of lignin in LCBM. It is easy to see from Table 2 that the degradation and reduction of ADL content after treatment of different LCBMs using different strains of fungi showed a wide range of differences, ranging from a loss of 9.40% to 84.59%. The only exception was Olagunju et al., who reported that using *T. versicolor* increased the ADL content of sorghum stover by 23.27% [62]. This is because *T. versicolor* does not selectively depolymerize lignin from sorghum straw but instead utilizes large amounts of hemicellulose for its growth metabolism. However, this does not mean that the nutrient availability of sorghum stover after solid-state fermentation with *T. versicolor* is reduced and needs to be further evaluated using in vitro rumen fermentation and other experiments. Lignin content may appear to increase due to analytical methods such as acid detergent lignin (ADL) may overestimate lignin if fungal melanins or modified lignin residues persist, and temporal dynamics whereby early-stage fungal colonization might show transient lignin accumulation due to incomplete degradation [49,63].

The CP content of LCBM after solid-state fermentation with WRF increased, ranging from 0.68% to 84.3%. The increase in CP content can be attributed to several reasons. Firstly, protein-containing mycelium is synthesized during solid-state fermentation, during which WRF utilize carbon as an energy source. Secondly, some fungi may be able to capture nitrogen and urea, which may occur under aerobic conditions. In addition, WRF secretes extracellular enzymes, which are essentially proteins. Finally, it has also been proposed that the increase in CP content is due to the consumption of carbon sources by WRF in solid-state fermentation, resulting in a higher percentage of CP. The reasons for the large differences in CP content increments are more complex and may be related to fermentation conditions and the suitability of WRF to LCBM. In other scholars’ and our previous experimental studies, LCBM after solid-state fermentation of LCBM produced more isoacids in the rumen in vitro fermentation experiments [34,61,67]. The increase in isoacids such as isobutyric, isovaleric, and valeric acids (BCVFA) can be attributed to the rise in a proportion of branched-chain amino acids (BCAAs) such as valine, leucine, and isoleucine, which are essential amino acids that can only be obtained through feed, in the LCBM after solid-state fermentation with WRF [68]. WRF solid-state fermentation of LCBM also produces large amounts of β-glucan [67,69]. β-glucan can increase ruminant intake of forage NDF, ADF, and low-quality rice straw [70,71]. In addition to β-glucan, secondary metabolites that may be produced after solid-state fermentation of LCBM through WRF that are beneficial to ruminants have not been explored, and further research in this area needs to be strengthened. RFV (relative feed value), RFQ (relative feed quality), and QI are all quality indices used to visually assess forage, and this review calculates the results of related studies to present a clearer picture of the quality changes in LCBM before and after solid-state fermentation with WRF. In short, the RFV, RFQ, and QI indices of LCBM after WRF solid-state fermentation were all enhanced. However, the magnitude of the enhancement varied greatly. For example, the RFV index, which is the most widely used index, was enhanced between 7.61% and 39.27% for wheat straw after solid-state fermentation with different fungi WRF. The main reason for this phenomenon is the difference in the depletion of easily digestible nutrients by different WRFs during solid-state fermentation.

### 5.2. Experimental Evaluation of Rumen In Vitro Fermentation of LCBM After Solid-State Fermentation

LCBM, after solid-state fermentation of WRF, enhances in vitro rumen fermentation in different ways (Table 3). The pH of rumen fluids was all down-regulated due to increased accessibility of rumen microorganisms to digestible nutrients as a result of lysis of the cell wall of LCBM, which was corroborated by TVFA (total volatile fatty acid) production, DMD (dry matter degradation), and T-Gas (total gas production). In other words, the rumen digestibility of LCBM after solid-state fermentation with WRF was increased. Rumen microbiota degrades woody fiber biomass into substances such as soluble sugars, which are further converted into VFA for energy metabolism, a vital energy donor for ruminants [72,73]. Datsomor et al., Niu et al., and Zuo et al. noted that maize stover, rice straw, bagasse, and wheat straw that underwent solid-state fermentation with different WRFs, when used in an in vitro fermentation experiment utilizing rumen fluid from different animal donors, increased T-Gas by 19.73% to 28.70% [33,46,48]. The digestion and transformation of nutrients by the rumen microbiota are accompanied by the production of various gases (CO_2_, CH_4_, and H_2_), and the increase in T-Gas can also be attributed to the increased rumen digestibility of LCBM after solid-state fermentation with WRF. BCVFA has been reported to be an essential growth factor for certain fiber-degrading bacteria in the rumen and has been shown to have a beneficial effect on increasing the activity of Rhodococcus yellows, Rhodococcus albicans, and filamentous succinate-producing bacteria [74]. This benefit may positively impact the efficiency of rumen fiber degradation in ruminants, consequently, feed efficiency [75,76]. As shown in Table 3, some of LCBM after solid-state fermentation of WRF increased the amount of BCVFA in the rumen. The use of fungi in solid-state fermentation of plants, which results in enhancement of plant proteins, is well described in the study of Wang et al. [77] The alteration of the amount of protein in LCBM after fermentation with WRF needs to be studied more intensively, particularly how the alteration of the amount of protein moderates the rumen microbial communities.

### 5.3. Experimental Evaluation of Rumen In Vivo Fermentation of LCBM After Solid State Fermentation

Compared to many experimental studies on other LCBM pretreatment means (air-blasted, crushed, silage, etc.), in vivo experimental reports on WRF solid-state fermentation LCBM appear extremely sparse. However, it is still possible to extract key information from the few studies demonstrating the value of WRF solid-state fermentation LCBM for applications [8]. Feeding rations containing LCBM after solid-state fermentation with WRF improves nutrient intake and nutrient digestibility in cows, goats, cattle, and sheep (Table 4). Improved nutrient digestibility may result in several gains, the most significant of which is, first and foremost, an increase in ruminant production performance. Sufyan et al. noted that the addition of *P. ostreatus* solid-state fermented wheat straw to dairy feed increased the apparent total tract digestibility of DM, OM, CP, NDF, and ADF by 8.14%, 8.21%, 14.01%, 15.57%, and 17.58%, respectively [39]. In the analysis of milk production performance, the milk yield of cows was increased by 6.63%, and the nutrient contents of milk fat, lactose, milk protein, and total milk solids were significantly increased by 5.10%, 7.02%, 3.67%, and 5.92%, respectively (Table 5). It is suggested that wheat straw, after solid-state fermentation of *P. ostreatus*, can enhance the performance of dairy cows and reduce the feed inputs for dairy cattle feeding. In addition, feeding oil palm fronds that have undergone solid-state fermentation with *LS-caju* improves the growth performance of goats [80]. Specifically, feeding *LS-caju* solid-state fermented oil palm leaves increased WG (weight grain), ADG (average daily gain), and G/F (gain-to-feed) ratio of goats by 4.05%, 5.41%, and 7.56%, respectively. Even more surprisingly, feeding solid-state fermented LCBM with WRF also improves health of ruminants. Xiang et al. feeding solid-state fermented corn straw by *L. edodes* to gastrointestinal nematodes-infected lambs reported improved lamb health performance through a decreased fecal egg count and increased packed cell volume [81]. Continuous improvement of ruminant growth, production performance, and maintenance of ruminant health performance are goals that the ruminant farming industry has been striving to achieve for a long time. In vivo, experimental studies in ruminants need to be further advanced to determine the necessity of utilizing LCBM after solid-state fermentation by WRF.

## 6. CH_4_ Emissions

Reducing CH_4_ emissions from ruminants is currently a central research proposition for reducing emissions from the livestock, as methane contributes more to global warming potential than other greenhouse gases; more than half of the methane emissions from livestock are produced by ruminants [85,86].

Sufyan et al. (2024) noted that solid-state fermentation of wheat straw with *P. ostreatus* reduced CH_4_ emissions during in vitro rumen fermentation in dairy cows from 29.1 mL/g OM to 26.1 mL/g OM [39]. In the study by Zhao et al. (2020), *F. filiformis* solid-state fermentation of corn stover reduced CH_4_ emissions from 30.39 mL/g DM to 24.36 mL/g DM in an in vitro fermentation using goat rumen fluid [55]. All of the above studies showed that LCBM, after solid-state fermentation by WRF, could be a novel CH_4_ inhibitor.

The ability of LCBM after solid-state fermentation with WRF to reduce CH_4_ emissions may be achieved through two essential pathways. Firstly, the reduction in rumen methane production may be related to lovastatin (LV), a secondary metabolite of WRF [87]. LV inhibits two key enzymes of rumen methanogens, HMG-CoA reductase and F420 coenzymes, which in turn inhibit the activity of rumen methanogens, reducing methane emissions [88]. A study by Jahromi et al. (2013) confirmed that LV-enriched rice straw significantly reduced rumen CH_4_ emissions [87]. Secondly, increasing propionic acid production in the rumen also appears to downgrade methane emissions, which is achieved by converting the production of CH_4_ from hydrogen utilized by methanogenic bacteria into the production of more propionic acid. Experimental studies by Sufyan et al. (2024) versus Zhao et al. (2020) corroborate this view [39,55].

## 7. Future Perspectives on the Limitations of WRF Solid-State Fermentation for LCBM Treatment

At a time when the global per capita arable land area is decreasing and environmental pressure continues to increase, the use of WRF for the solid-state fermentation LCBM to enhance the efficiency of LCBM for ruminants has a promising future. The solid-state fermentation of LCBM using WRF has broad application and research value. Overall, solid-state fermentation of LCBM using WRF can effectively enhance its nutritional quality. In order for the technology of WRF solid-state fermentation of LCBM to be further applied in the ruminant farming industry, further research needs to be conducted.

The awareness of human concern for food safety and animal breeding is increasing. The application of WRF in human food has been well accepted. However, limited research has been conducted to evaluate the safety of solid-state fermented LCBM using WRF. Conducting experimental studies on safety is conducive to the further acceptance and advancement of the ruminant industry’s application of WRF solid-state fermented LCBM.

Screening studies for WRFs using high-yield LCBM (bagasse, wheat straw, rice straw, corn straw, etc.) require further investigation. The screening principles consist of two main aspects. Firstly, the WRF needs to fully occupy a dominant ecological niche or a relatively dominant one in a non-sterile substrate. Secondly, the screening of WRF for efficient solid-state fermentation effect is carried out for a specific LCBM. Finally, targeted modification studies of WRF can be done through gene editing and other means.

The challenge of the growth of non-target microorganisms hindering the effective application of WRF has been one of the greatest difficulties in utilizing WRF in various fields, and a detailed review of utilizing WRF under non-sterile conditions has been conducted by Svobodova et al. [89]. Most of the studies on solid-state fermentation of LCBM by WRF as a feed component for ruminants have been based on sterilized conditions, which are unfeasible mainly for economically orientated ruminant industry. At the same time, the energy consumption caused by sterilization goes against the basic principle of ‘greening’ the use of WRF solid-state fermented LCBM. Synthetic microbial communities may be a potential solution for the application of WRF under non-sterile conditions. Chen et al. isolated microorganisms from maize stover under different stockpiling conditions using a ‘top-down’ principle and subsequently constructed a synthetic microbial community [90]. The results showed that the synthetic microbial community’s cellulose, hemicellulose, and lignin degradation rates of corn stover were 34.91%, 45.94%, and 23.34%, respectively. Another principle of synthetic microbial communities is known as the ‘bottom-up.’ In short, microbial communities that can be assembled based on metabolite cross-feeding relationships are analyzed via genomic and other means, such as strain characteristics and nutritional requirements [91]. Forming a synthetic microbial community with WRF as the main effector microorganism and other microorganisms (fungi and bacteria) to occupy the dominant ecological niche under non-sterile conditions is theoretically feasible. In addition, screening for beneficial substances that can promote the early and rapid colonization of WRF is also a significant direction for future work. However, most of the current reports have only focused on screening beneficial substances to promote the degradation efficiency of WRF.

In vivo experiments are the most effective means of characterizing the animal’s availability and value of feeds. Few in vivo experiments have been conducted to characterize the efficacy of solid-state fermentation of LCBM with WRF. In particular, there is a gap in the reports on the effects on animal body metabolism and rumen microbiota. In addition, carrying out economic benefit accounting (feed cost conversion ratio, etc.) is recommended when conducting in vivo animal experiments.

Researching high-value-added bio-products is also a viable way to utilize LCBM through solid-state fermentation with WRF. LCBM can be combined with other agricultural by-products to enrich the production of a specific metabolite that substantially increases animal growth performance and production performance, using the previously mentioned LV, β-glucan, etc. Carrying out high-value-added bioproducts facilitates the centralized utilization of LCBM. In addition, the economic burden and energy pressure caused by the pretreatment of LCBM using autoclaving and other means can be compensated for by developing high-value-added bioproducts.

Whether WRF solid-state fermented LCBM can reduce the environmental problems caused by LCBM abandonment is unanswered. In addition, it is still unknown whether the whole process of feeding WRF solid-state fermented LCBM to ruminants impacts carbon footprint. This part of the study is recommended in the future to promote the research and application of WRF solid-state fermented LCBM.

## 8. Conclusions

In conclusion, the selection of WRF solid-state fermentation of LCBM is an effective means to efficiently improve the utilization efficiency of LCBM by ruminants. However, the current solid-state fermentation of LCBM using WRF still needs to overcome practical application challenges such as the need for a sterile environment and WRF-substrate suitability. Further future adoption will contribute to the global adoption of WRF solid-state fermentation LCBM if the current technical limitations can be overcome.

## Figures and Tables

**Table 2:** WRF solid-state fermentation LCBM front and rear nutrition chemical composition and changes in the nutritional index (%).

Strain/Straw	DM	CP	NDF	ADF	ADL	HC	CL	OM	RFV	RFQ	QI	Ref.
Wheat straw												
*P. ostreatus*	−19	+25.8	−15.6	−24.3	−38.8-	−38.2	−21.4	−20.6	+8.51	+4.75	+3.31	[64]
*I. lacteus*	−22.34	+25.5	−14.00	−4.00	−43.08	−46.03	−20.13	−22.2	+15.40	+24.51	+22.19	[46]
*P. eryngii*	−12.15	+16.35	−5.2	−2.32	−20.36	−14.57	+1.32	−12.46	+7.61	+16.18	+13.89	[65]
*Pleurotus florida*	−8.69	+49.73	−11.54	−15.13	−10.40	−2.13	−16.15	−3.09	+30.02	+33.52	+22.16	[66]
*P. chrysosporium*	−45.17	+34.43	−28.26	−28.04	−24.00	−61.00	−61.90	−47.96	+39.27	+48.77	+45.54	[46]
Rice straw												
*P. ostreatus*	−5.92	+60.88	−16.53	−10.5	−31.89	−28.1	−6.75	−9.63	+22.09	+27.77	+25.44	[48]
*C. eriporiopsis*	-	+22.93	−22.83	+3	−84.59	−58.83	+12.32	−3.51	+21.48	+35.88	+33.20	[52]
*L. edodes*	-	+20.85	−20.71	+1.73	−73.61	−48.60	+6.53	−2.74	+21.49	+34.60	+31.84	[52]
*P. eryngii*	−4.50	+19.30	−21.00	−7.70	−41.10	−43.00	−2.90	−4.90	+36.67	+31.05	+19.34	[51]
*P. chrysosporium*	−13.14	+22.83	−29.74	−31.56	−24.26	−19.83	−23.93	−22.68	+35.74	+43.73	+40.45	[49]
Corn stover												
*P. ostreatus*	−10.50	+66.19	−18.35	−18.13	−44.67	−18.52	−9.07	−1.92	+22.67	+27.16	+24.62	[53]
*P. eryngii*	+0.24	+31.21	−15.91	−16.72	−33.12	−15.03	-	−3.90	+21.74	+25.83	+23.65	[55]
*F. filigormis*	+0.31	+33.99	−21.04	−13.54	−20.14	−29.17	-	−2.87	+25.53	+32.42	+29.91	[55]
*L. edodes*	+0.61	+36.29	−17.79	−12.14	−29.88	−23.90	-	−2.80	+22.01	+26.47	+24.26	[55]
*P. diamor*	-	+33.19	−7.00	−5.80	−15.03	−9.74	−6.43	−7.52	+11.24	+11.80	+10.55	[56]
Other LCBM												
*L. edodes*/Rape straw	−16.8	+22.82	−17.0	−14.3	−9.40	−24.9	−15.7	−17.2	+30.34	+38.51	+34.97	[57]
*P. ostreatus*/Bagasse	−15.10	+84.3	−31.70	−26.50	−41.50	−41.80	−23.00	+23.30	+29.64	+44.19	+38.92	[58]
*P. citrinopileatus*/ Grape Stalks	−33.96	+51.81	−2.93	−8.37	−19.29	−36.75	−2.51	−4.66	+5.53	+8.94	+7.80	[59]
*T. versicolor*/Sorghum	+3.99	+28.50	−23.73	+23.19	+20.32	−64.49	+23.65	+3.10	+13.61	+26.05	+24.07	[62]
*P. chrysosporium*/White tea straw	−5.03	+0.68	−7.89	−13.98	−25.64	+2.47	−6.47	−0.06	+11.15	+11.64	+11.19	[61]

Note: “-” indicates that the data were not obtained or could not be calculated. *P. ostreatus*, *Pleurotus ostreatus*; *T. versicolor*, *Trametes versicolor*; *C. subvermispora*, *Ceriporiopsis subvermispora*; *L. edodes*, *Lentinula edodes*; *I. lacteus*, *Irpex lacteus*; *P. eryngii*, *Pleurotus eryngii*; *P. chrysosporium*, *Phanerochaete chrysosporium*; *P. diamor*, *Pleurotus djamor*; *Pleurotus sajor-caju*; *P. citrinopileatus*, *Pleurotus citrinopileatus*.

**Table 3:** Positive and negative effects of feeding rations containing LCBM after solid-state fermentation of WRF on nutrient In vitro fermentation (%).

Strain	LCBM	Animal	pH	NH_3_-N	T-Gas	TVFA	A-Acid	P-Acid	Ib-Acid	B-Acid	Iv-Acid	V-Acid	DMD	Ref.
*P. sajor-caju*	Barely straw	Yak	-	+23.70	-	+7.05	+3.00	−3.39	-	+6.81	-	-	+11.88	[77]
*I.* *lacteus*	Corn straw	Cow	-	-	-	+7.28	−2.18	+10.95	−12.63	−5.79	−7.63	−16.67	-	[78]
*P. ostreatus*	Corn straw	Cow	-	-	-	+0.93	−1.25	+2.71	+17.15	−4.06	+6.14	−4.17	-	[78]
*L. edodes*	Corn straw	Goat	−2.47	+19.04	+19.73	+12.64	−0.88	+0.56	-	+4.40	-	+14.43	+16.53	[55]
*P.* *chrysosporium*	Rape straw	Cattle	-	-	-	+18.68	+23.31	-	-	-	-	11.90	-	[57]
*P. ostreatus*	Rice straw	cow	−0.31	-	+29.39	+36.40	−6.62	+22.24	+35.09	−27.64	+78.43	+68.63	+17.59	[48]
*P. cornucopiae*	Grape pomace	Lamb	-	-	-	+22.61	-	-	-	-	-	-	+11.08	[79]
*P. ostreatus*	Sugarcane bagasse	Cow	-	-	+28.00	-	-	-	-	-	-	-	+20.80	[58]
*I.* *lacteus*	Wheat straw	Cattle	−1.01	+14.32	+28.70	+5.39	−2.11	+10.48	−3.17	−8.86	−5.99	−6.86	-	[46]

Note: “-” indicates that data were not obtained or could not be calculated. T-Gas, Total Gas production; TVFA, Total of volatile fatty acid; A-acid, acetic acid; P-acid, propanoic acid; IB-acid, isobutyric acid; B-acid, butyric acid; Iv-acid, isovaleric acid; V-acid, valeric acid; DMD, dry matter degradation; *P. ostreatus*, *Pleurotus ostreatus*; *L. edodes*, *Lentinula edodes*; *I. lacteus*, *Irpex lacteus*; *P. eryngii*, *Pleurotus eryngii*; *P. chrysosporium*, *Phanerochaete chrysosporium*; *P. diamor*, *Pleurotus djamor*; *P. sajor-caju*, *Pleurotus sajor-caju*.

**Table 4:** Positive and negative effects of feeding rations containing LCBM after solid-state fermentation of WRF on nutrient intake, nutrient digestion in ruminants (%).

Strain	LCBM	Animal	DMI	OMI	CPI	NDFI	ADFI	DMAD	OMAD	CPAD	NDFAD	ADFAD	Ref.
*P. ostreatus*	Wheat straw	Cow	+5.94	+4,72	+6.57	−2.41	−2.27	+8.14	+8.21	+14.01	+15.57	+17.58	[39]
*P. pulonarius*	Emp fruit bunch	Goat	−2.34	-	+2.91	+3.18	+10.05	+10.86	-	+8.66	+13.82	+21.12	[82]
*P. sajor-caju*	Oil palm frond	Goat	−3.74	+0.96	-	−12.5	+3.23	+10.13	+10.03	+11.98	+10.68	+29.90	[83]
*P. sajor-caju*	Oil palm frond	Goat	−2.13	−1.19	+0.92	−5.73	−4.60	+5.07	+5.35	-	-	-	[80]
*P. chrysosporium*	Cocoa pod	Cow	+11.19	+43.20	+6.09	+5.99	+5.94	+9.82	+18.26	+24.28	+30.55	+13.57	[84]
*Crinipellis* spp.	Wheat straw	Cattle	+14.37	+11.54	-	-	-	+9.63	+9.94	+7.71	+14.10	+28.65	[82]

Note: “-” indicates that data were not obtained or could not be calculated. DMI, dry matter intake; OMI, organic matter intake; CPI, crude protein intake; NDFI, neutral detergent fiber intake; ADFI, acid detergent fiber intake; DMAD, apparent total tract digestibility of dry matter; OMAD, apparent total tract digestibility of organ matter; CPAD, apparent total tract digestibility of crude protein; NDFAD, apparent total tract digestibility of neutral detergent fiber; ADFAD, apparent total tract digestibility of acid detergent fiber; *P. ostreatus*, *Pleurotus ostreatus*; *C. subvermispora*, *Ceriporiopsis subvermispora*; *P. chrysosporium, Phanerochaete chrysosporium; Pleurotus sajor-caju*.

**Table 5:** Positive and negative effects of feeding rations containing LCBM after solid-state fermentation with WRF on milk yield and milk components performance of ruminants.

Strain	LCBM	Animal	Milk Yield	FCM Yield	Fat	Lactose	Protein	Total Solid	Ref.
*P. ostreatus*	Wheat straw	Cow	+6.63	+5.81	+5.10	+7.02	+3.67	+5.92	[39]
*P. pulonarius*	Emp fruit bunch	Goat	−1.43	-	+4.87	−0.65	+5.71	+1.75	[82]

Note: “-” indicates that data were not obtained or could not be calculated. FCM, fat corrected milk. *P. ostreatus*, *Pleurotus ostreatus*; *P. pulonarius, Pleurotus pulmonarius*.
